# Integrated T cell cytometry metrics for immune-monitoring applications in immunotherapy clinical trials

**DOI:** 10.1172/jci.insight.160398

**Published:** 2022-10-10

**Authors:** Dimitrios N. Sidiropoulos, Genevieve L. Stein-O’Brien, Ludmila Danilova, Nicole E. Gross, Soren Charmsaz, Stephanie Xavier, James Leatherman, Hao Wang, Mark Yarchoan, Elizabeth M. Jaffee, Elana J. Fertig, Won Jin Ho

**Affiliations:** 1 Johns Hopkins University School of Medicine, Baltimore, Maryland, USA.; 2 Johns Hopkins Convergence Institute, Sidney Kimmel Comprehensive Cancer Center, Baltimore, Maryland, USA.; 3 Johns Hopkins Bloomberg Kimmel Institute for Immunotherapy and; 4 Department of Oncology, Sidney Kimmel Comprehensive Cancer Center, Johns Hopkins Medicine, Baltimore, Maryland, USA.; 5 Department of Applied Mathematics and Statistics, Johns Hopkins University, Baltimore, Maryland, USA.; 6 Department of Biomedical Engineering, Johns Hopkins University School of Medicine, Baltimore, Maryland, USA.

**Keywords:** Immunology, Oncology, Bioinformatics, Cancer immunotherapy, Statistics

## Abstract

Mass cytometry, or cytometry by TOF (CyTOF), provides a robust means of determining protein-level measurements of more than 40 markers simultaneously. While the functional states of immune cells occur along continuous phenotypic transitions, cytometric studies surveying cell phenotypes often rely on static metrics, such as discrete cell-type abundances, based on canonical markers and/or restrictive gating strategies. To overcome this limitation, we applied single-cell trajectory inference and nonnegative matrix factorization methods to CyTOF data to trace the dynamics of T cell states. In the setting of cancer immunotherapy, we showed that patient-specific summaries of continuous phenotypic shifts in T cells could be inferred from peripheral blood–derived CyTOF mass cytometry data. We further illustrated that transfer learning enabled these T cell continuous metrics to be used to estimate patient-specific cell states in new sample cohorts from a reference patient data set. Our work establishes the utility of continuous metrics for CyTOF analysis as tools for translational discovery.

## Graphical abstract

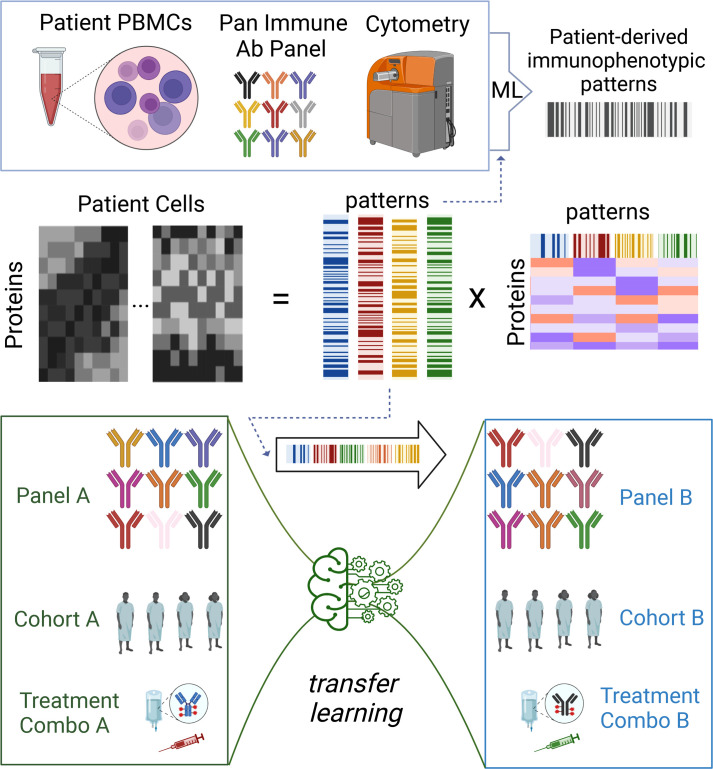

## Introduction

Although cancer immunotherapies, such as immune checkpoint blockade, have been established as an effective treatment modality for several cancer types, the majority of solid tumor patients still do not respond (1). In the setting of numerous ongoing immunotherapy clinical trials, understanding how each patient’s immune system is configured at baseline and changes throughout the course of treatment is pivotal to identifying strategies that can further improve immunotherapeutic efficacy. Thus, methods for evaluating the immunologic states of large numbers of patients and cell-scale dynamics within each patient, even in the setting of negative clinical responses in a cost-effective, scalable manner are critically warranted.

Cytometry by TOF (CyTOF) is a high-parameter cytometry tool that has been used in several immunotherapy studies to report immune composition in peripheral blood and determine overall systemic responses to therapy (2–5). The appeal of this technology stems from its use of antibodies conjugated to isotopically enriched heavy metals, overcoming multiple challenges encountered in fluorescence-based cytometry (6) and enabling the simultaneous measurement of more than 40 parameters in a relatively cost-effective manner in comparison with other single-cell profiling tools (7). The high-dimensional nature of these data provides the ability to conduct detailed subtyping of cells at the protein level to monitor phenotypic shifts within those cells with high fidelity.

CyTOF antibody panels have been successfully designed to recapitulate discrete cell types from all major immune lineages (2, 3, 8–10). Many clinical applications then adopt analysis pipelines that prioritize the frequency of specific cellular subpopulations within the data set to distinguish immunologic efficacy (4, 11). Still, the function and state of each of these cells further contributes to the ultimate clinical benefit of immunotherapeutic response (12). Whereas these discrete cell-type characterizations enable immunological observations consistent with previous flow-based studies, capturing the entirety of cell states in a given sample as they undergo continuous phenotypic changes can better reflect the functional dynamics within the overall immunological context. For example, characterizing T cells as they transition from activated to exhausted states or effector to memory states is particularly relevant to cancer immunotherapy, because checkpoint immunotherapy focuses on reinvigorating exhausted T cells and promoting T cell activation. Advancements in computational pipelines for single-cell analysis have contributed alternative machine-learning frameworks, including notable trajectory inference and matrix factorization approaches, that fully leverage the multiparameter measurements to monitor cell-state transitions in these data sets (13–17). Specifically, studies leveraging single-cell RNA-Seq (scRNA-Seq) coupled with unsupervised clustering and trajectory inference methods have established that many cellular phenotypes often exist in continuums (12, 18, 19). These studies have shown that because biological systems operate dynamically, restricting cell-type definitions to canons at the transcriptional level may prevent us from identifying unique or understudied cellular populations (15, 20). Indeed, previous studies have leveraged trajectory algorithms, e.g., TRACER and Monocle3, in mass cytometry analysis to distinguish cellular states along biological continuums in various contexts (21, 22), but more work is needed to establish the translational utility of such approaches. Herein, we sought to determine whether continuous metrics derived from CyTOF-based profiles can be utilized as integrated biomarkers of T cell states and thus applied as measures of immunotherapy response in clinical data sets.

To derive continuous metrics, we applied 2 methods in parallel: we reduced CyTOF proteomic data dimensions to infer pseudotemporal immunological trajectories and employed a nonnegative matrix factorization (NMF) method, Coordinated Gene Association in Pattern Sets (CoGAPS) (23, 24), to generate continuous weights to represent continuous phenotypic states of T cells. To first benchmark these metrics, we tested whether the derived continuous CyTOF metrics could recapitulate T cell exhaustion states, an important functional feature to be monitored in the setting of cancer immunotherapy. Given the well-established observations of T cell exhaustion in HIV (25, 26), we first applied our pipeline to a publicly available CyTOF data set of 32 markers from patients with HIV and healthy controls to derive continuous T cell metrics. Both pseudotemporal and CoGAPS-based signatures accurately recapitulated the progression of HIV-associated T cell exhaustion biology. We then applied this approach to data sets generated in immunotherapy clinical trials from diverse diseases, such as in pancreatic ductal adenocarcinoma (PDAC), hepatocellular carcinoma (HCC), and melanoma. We again computed integrated functional CyTOF metrics from these trajectories and correlated them with disease states and treatment outcomes. Finally, we demonstrate the ability of transfer learning to project disparate data sets into these CyTOF-derived single-cell proteomic metrics to compute continuous T cell states in new clinical samples. Our work offers a functional framework for immune system proteomics that can empower biomarker discovery and analysis in cancer immunotherapy.

## Results

### Single-cell proteomic pseudotemporal ordering of CD8^+^ T cells in patients with HIV inferred a trajectory of T cell exhaustion and described patient-level T cell states.

**Figure 1:**
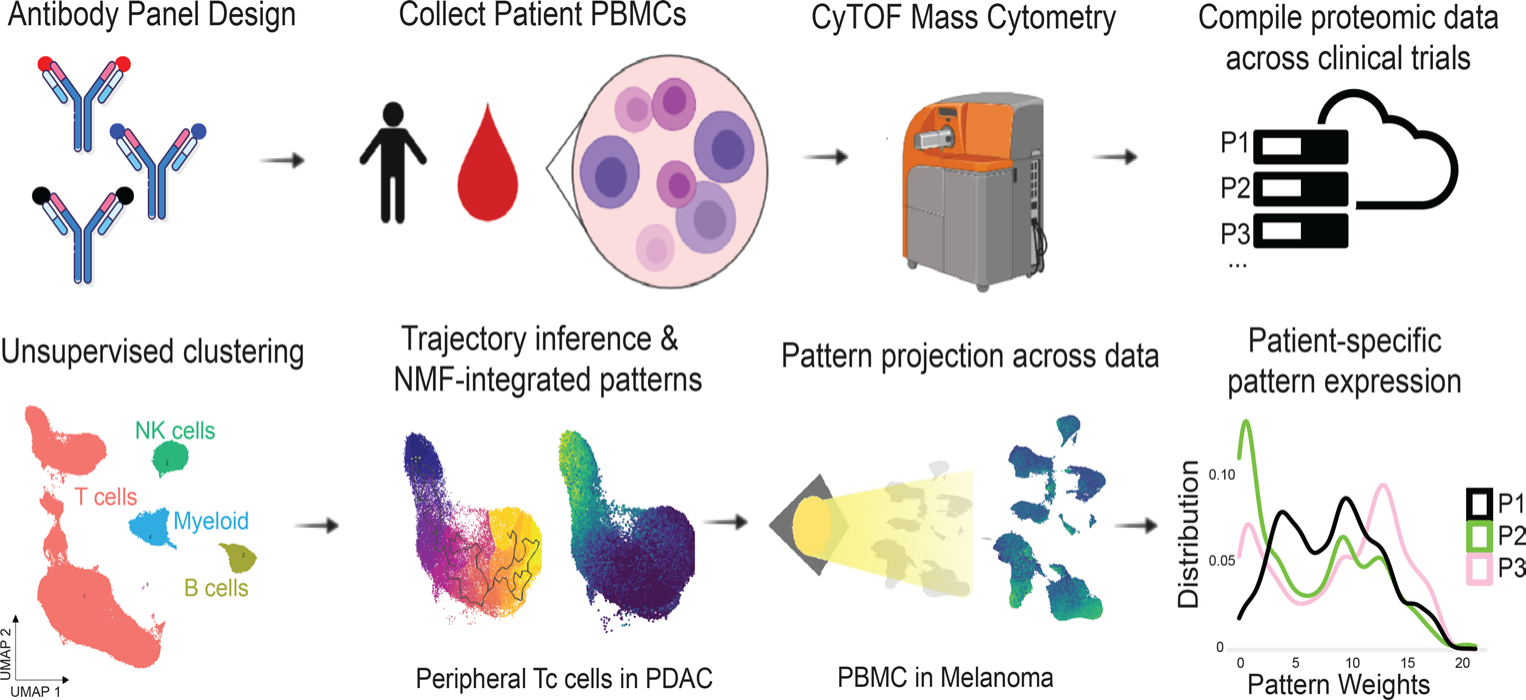
Protein quantification of T cell states at the single-cell level. Workflow illustrating patient sample collection to data generation and computation of integrated metrics that can be projected across data sets.

Trajectory inference and pseudotime methods have demonstrated the ability to compute measurements of cell-state transitions along a biological process in scRNA-Seq data (16). We aimed to construct a quantitative metric of continuous phenotypic shifts associated with T cell activation and the transition from T effector to T memory cells from CyTOF data as functional biomarkers in patient immune responses. Using our framework a distribution of patient-specific T cell states to use as biomarkers (Figure 1). Given the well-established biology of T cell exhaustion in HIV infection, we first benchmarked the ability to use patient-specific summaries of T cell states based upon pseudotime analysis of publicly available CyTOF data for PBMCs of 24 treated and untreated patients with HIV and healthy controls (25).

**Figure 2:**
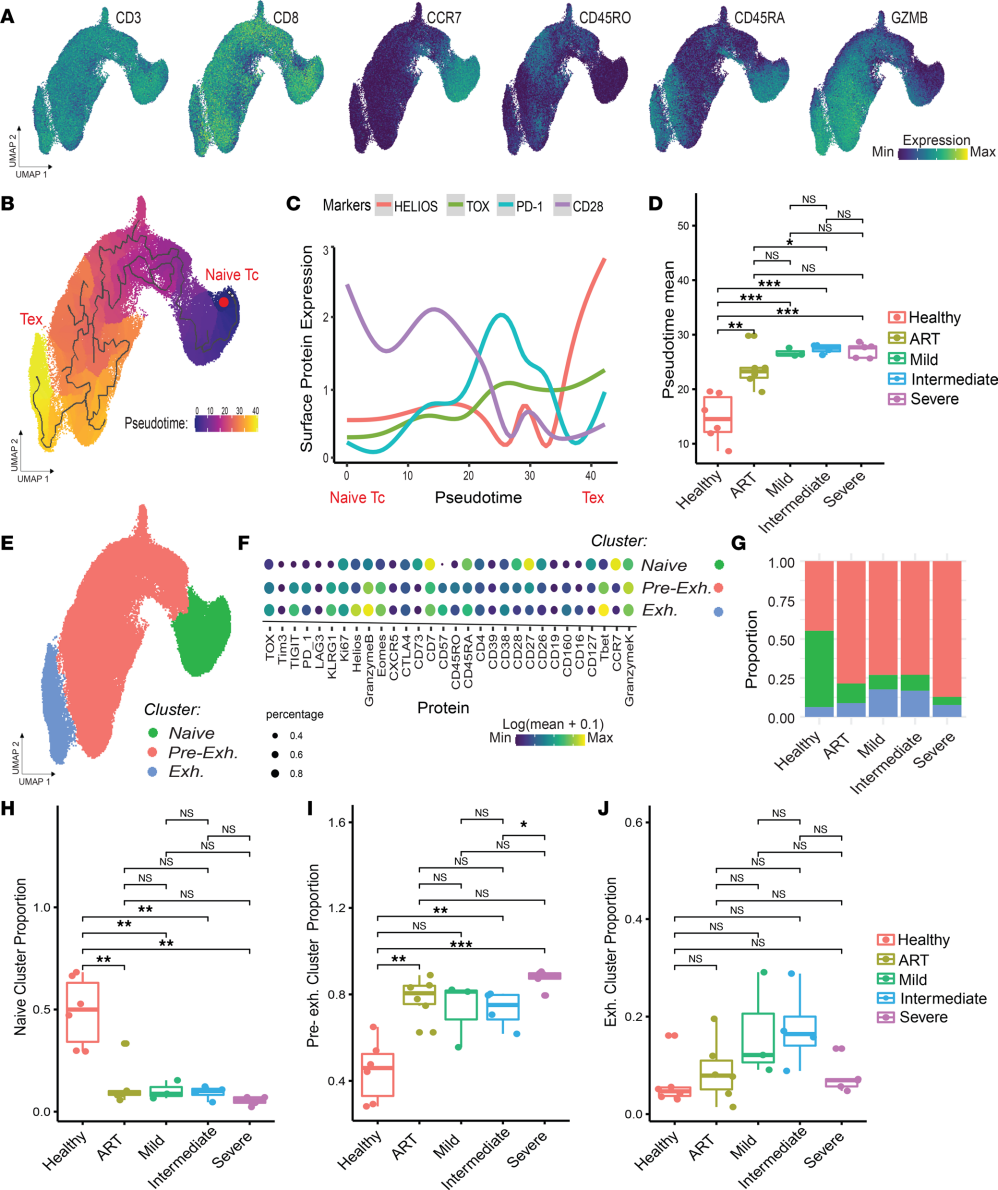
Protein quantification of T cell states at the single-cell level. (**A**) Protein expression of representative canonical T cell markers present in the HIV CyTOF panel. (**B**) Pseudotime trajectory computed using naive CD8^+^ T cells (Tc) as origin. (**C**) Fitted model of surface protein expression of cells ordered by pseudotime from naive Tc to exhausted (Tex), demonstrating transient expression of activation and exhaustion. (**D**) Pseudotime mean by patient across HIV status and treatment. Statistical significance was determined by group mean comparison *t* tests. (**E**) Clustering analysis projected on UMAP. (**F**) Dot plot of mean expression by cluster. (**G**) Bar plot showing compositionality by condition. (**H**–**J**) Box plots of proportion of each cluster by patient, grouped by condition. Statistical significance was determined by group mean comparison *t* tests. Statistically significant *P* values adjusted for multiple comparisons (Holm-Bonferroni) are shown as follows: *P < 0.05; **P < 0.01; ***P < 0.001.

To define our T cell states, we ordered cells by selecting a naive (CCR7^+^CD45RA^+^) phenotype as the trajectory’s origin. We computed pseudotime weights of developmental T cell-state transitions, wherein 0 was representative of the selected origin and increasing weights corresponded with progression through the trajectory (Figure 2, A and B). Replicating the findings of Bengsch et al. (25), we found that the pseudotime trajectory associated with T cell exhaustion states defined by specific markers of T cell exhaustion, such as TOX and HELIOS, increased along pseudotime, while naive and activation markers, such as CD28, decreased (Figure 2C and Supplemental Figure 1). We then formed a patient-specific metric of expected T cell exhaustion using the mean pseudotime weight. Comparison of the pseudotime-based estimation of T cell exhaustion in each patient between healthy controls and infected patients in a *t* test demonstrated that healthy controls had significantly lower pseudotime compared with infected patients, indicative of a less exhausted profile, regardless of disease severity (*t* test, *P* < 0.001; Figure 2D). We also found that patients on antiretroviral therapy (ART) had overall intermediate pseudotimes compared with untreated patients and healthy controls, suggestive of a lingering exhaustion profile. Thus, these pseudotime trends corresponded with known HIV-associated T cell biology, reflecting decreased functionality consistent with increased expression of exhaustion-specific markers in T cells from patients with HIV, even those on ART with undetectable viral loads (27).

In parallel with pseudotime analysis, we also tested how clusters of CD8^+^ T cells distinguished by Leiden clustering can be used to compare the profiles across the different HIV groups. We identified 3 distinct clusters, one of which we annotated as “naive” (CCR7^+^) and the other 2 as “preexhaustion” and “exhausted” (HELIOS*^+^*, TOX^+^) based on their protein expression profiles (Figure 2, E and F). As expected, the proportion of cells belonging to the naive CD8^+^ T cell cluster was consistently lower in HIV-infected groups compared with that in healthy controls (*t* test, *P* < 0.001, Figure 2, G and H), whereas the preexhaustion CD8^+^ T cell cluster was significantly more abundant in the ART, intermediate, and severe groups (*t* test, *P* < 0.001) but not in the mild group (Figure 2, G and I). Interestingly, in this discrete clustering analysis, no significant differences were observed in the exhausted CD8^+^ T cell cluster (Figure 2J). In contrast to the single-cell trajectory presented above, which employed a data-wide exploration of continuous and progressive biological trends, the clustering analysis suggests that relying on clusters alone may result in an inconsistent or unnecessarily skewed interpretation of the data by relying on discrete measures, underestimating differences in certain populations while overestimating in others.

### CoGAPS patterns in CyTOF data generated integrated patterns of protein expression programs that also reflected T cell states at the patient level.

**Figure 3:**
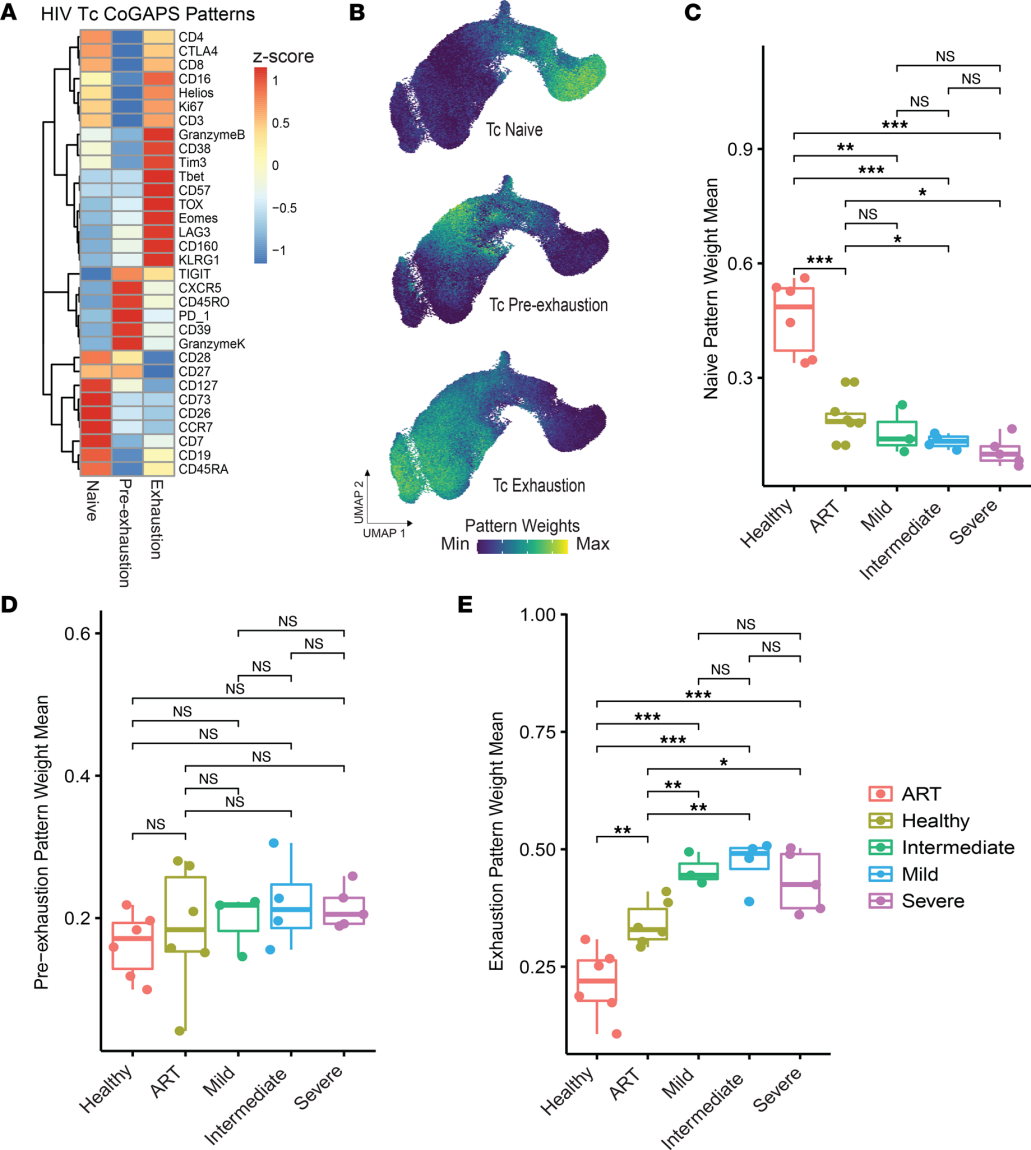
NMF can reveal biologically descriptive patterns of protein expression in CyTOF data. (**A**) Heatmap showing relative expression of protein markers in patterns generated using CoGAPS. (**B**) CD8^+^ T cells from the HIV data set represented in a UMAP overlaid with pattern weights. (**C**) Naive pattern–, (**D**) preexhaustion-, and (**E**) exhaustion-related pattern weight means by patient across conditions. Statistical significance was determined by group mean comparison *t* tests. Statistically significant *P* values adjusted for multiple comparisons (Holm-Bonferroni) are shown as follows: *P < 0.05; **P < 0.01; ***P < 0.001.

To compute continuous integrated protein metrics that enhance the resolution of T cell trajectory inference, which may span multiple cell states, while replacing discrete classifications in clustering analyses, we applied CoGAPS (23, 24) to CyTOF data. CoGAPS infers patterns of biological activity by identifying coregulated sets of proteins and decomposing an expression matrix into 2 sets of factors: cell-level pattern weights, ranging from 0 to a maximum weight of 1, and their corresponding protein-level amplitudes. In the same HIV data set described above, we computed 3 patterns from the protein expression that we annotated as naive, preexhaustion, and exhaustion based on the average pattern weights for each CyTOF panel marker (Figure 3A). These patterns mirrored the pseudotemporal trajectory inferred in the uniform manifold approximation and projection (UMAP) embedding described above but provided greater resolution of distinct T cell states without requiring either trajectory inference or the assumption of monotonic changes along that trajectory (Figure 3B). We compared pattern weight means across patients and found that naive pattern weights were significantly higher in healthy controls compared with ART-treated patients with HIV (*t* test, *P* < 0.01) and untreated patients (*t* test, *P* < 0.001) (Figure 3C). ART-treated patients also had significantly higher naive pattern weights compared with patients with intermediate and severe disease (*t* test, *P* < 0.05) but not compared with those with mild disease. Preexhaustion pattern weights indicated no differences among patients across groups (Figure 3D). Exhaustion pattern weights, however, mirrored the pseudotime-based results (Figure 3E). Specifically, healthy control patients exhibited significantly lower exhaustion pattern weights compared with patients with HIV (*t* test, *P* < 0.01, ART; *P* < 0.001, untreated). ART-treated patients also had significantly lower exhaustion pattern weights compared with untreated patients (*t* test, *P* < 0.001).

Similar to marker coexpression patterns identified from CoGAPS, analysis of single markers identified significant differences between patient groups. However, this was only observed for some markers, such as CD28 and TOX, but not for others, such as PD-1 and HELIOS (Supplemental Figure 2). This demonstrates that integrated T cell patterns can highlight both drivers of differential phenotypes as well as coexpressed or coregulated genes with a contribution to phenotype that might be missed at the single-gene level. Overall, these results provide a proof of concept that integrated metrics of patient-specific T cell states derived from pseudotime weights or CoGAPS patterns based on mass cytometry profiling can be employed to evaluate immunological contexts.

### CyTOF-based integrated T cell metrics can be directly used to describe clinically meaningful attributes in patients with PDAC treated with ipilimumab and GVAX.

**Figure 4:**
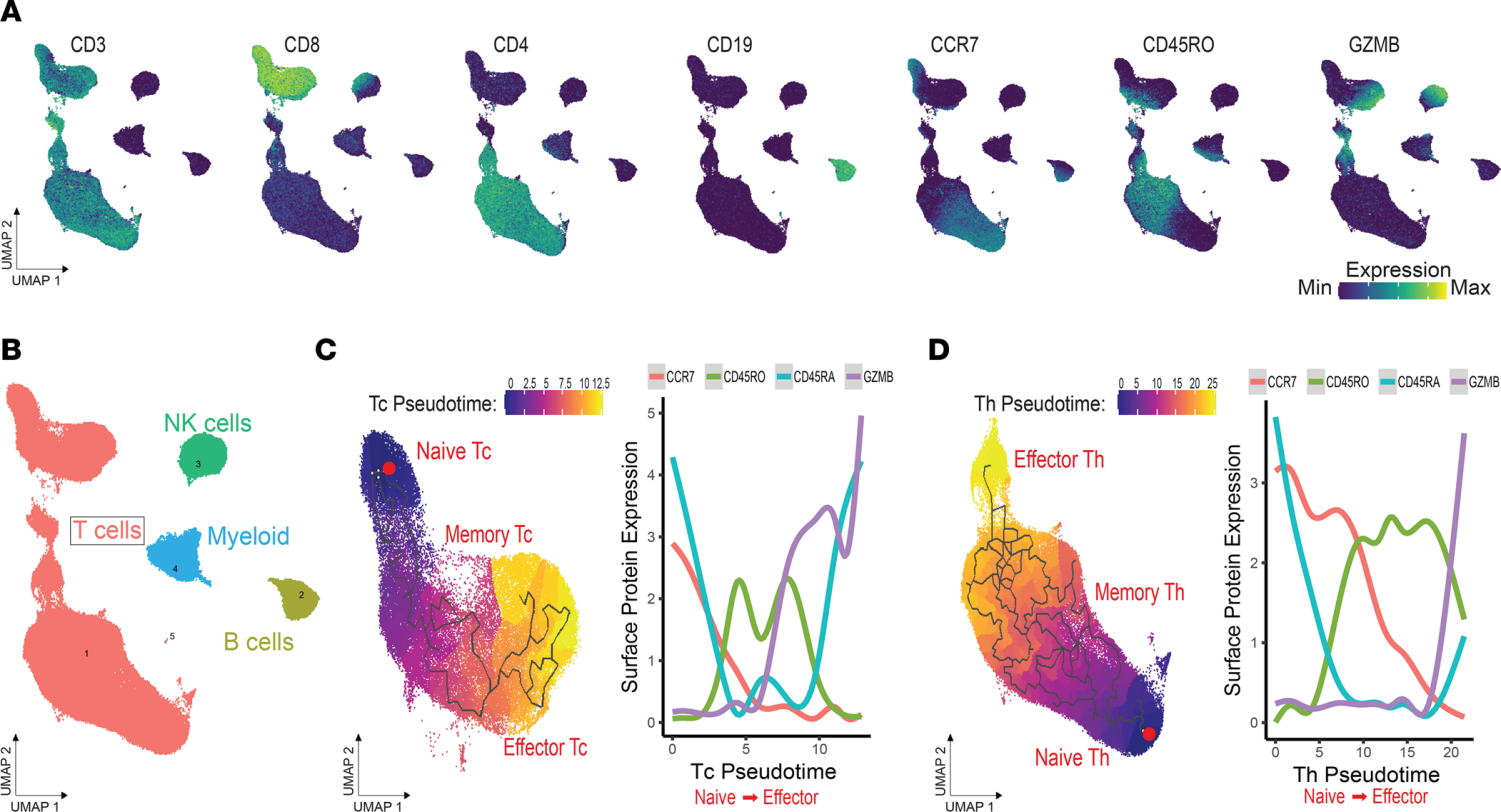
CyTOF integrated metrics recapitulate T cell differentiation from naive to effector phenotypes in peripheral blood of patients receiving cancer immunotherapy. (**A**) UMAPs colored by protein expression canonical markers representing major immune lineages in PBMCs from patients with PDAC undergoing ipilimumab and GVAX combination immunotherapy. (**B**) Annotated UMAP. (**C** and **D**) Pseudotime trajectory and fitted models of protein expression by pseudotime in CD8^+^ and CD4^+^ T cells, respectively.

**Figure 5:**
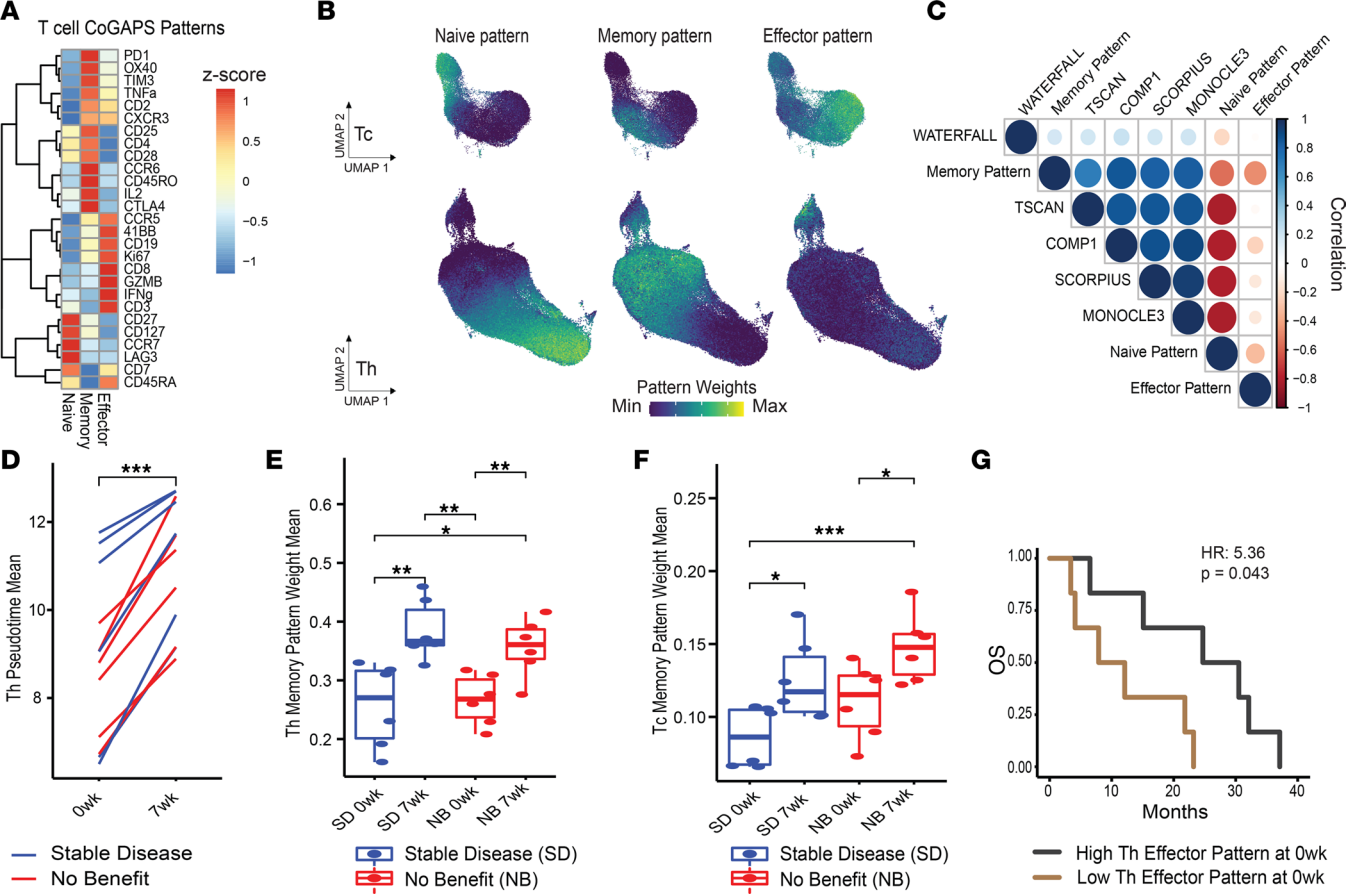
Integrated CyTOF metrics generated using CoGAPS have clinical utility. (**A**) Heatmap of relative marker protein expression in NMF patterns generated by CoGAPS in CyTOF data in peripheral blood T cells from patients with PDAC. (**B**) CD8^+^ Tc and CD4^+^ Th cell UMAPs colored by pattern weights. (**C**) Correlations between pseudotime methods and NMF patterns in Tc cells. (**D**) Mean Th cell pseudotime by patient at baseline and 7 weeks of treatment with ipilimumab and GVAX. (**E** and **F**) Memory pattern weight means by patients across time points and treatment response (SD, stable disease; NB, no benefit) in Th cell and Tc cells, respectively. (**G**) Comparison of overall survival (OS) by months in patients with PDAC with high Th cell effector pattern weight mean versus low Th cell effector pattern mean. Statistical significance was determined by (**D**) group mean comparisons, paired *t* tests for line plot; (**E** and **F**) unpaired *t* tests in box plots; and (**G**) Cox proportional hazards regression model in survival plot. Statistically significant *P* values adjusted for multiple comparisons (Holm-Bonferroni) are shown as follows: *P < 0.05; **P < 0.01; ***P < 0.001.

In addition to comparing T cell states in general, we aimed to determine the translational utility of CyTOF-based T cell metrics for individual patients defined by both trajectory inference and NMF using a time-course data set in the context of cancer immunotherapy. Specifically, we hypothesized that our CyTOF analysis approach could identify continuous Th cell and cytotoxic T (Tc) cell trajectories of antigen experience, including naive, memory, and effector T cell states, during immunotherapy. We previously generated CyTOF data using PBMCs from patients with PDAC treated with ipilimumab, a checkpoint immunotherapy targeting CTLA-4, and a GM-CSF–secreting allogeneic pancreatic cancer vaccine (GVAX). PBMCs were profiled at baseline and 7 weeks after treatment to monitor systemic immune responses across time (28). As done for the HIV data set, we first performed dimension reduction on the protein level to generate a UMAP representation of the CyTOF data (Figure 4A). The pan-immune panel used in the study yielded 4 distinct clusters belonging to T cells, NK cells, B cells, and myeloid cells (Figure 4B). Next, we subsetted Tc and Th cells and computed separate immune pseudotemporal trajectories (Figure 4, C and D). Again, ordering the cell trajectories by selecting naive cells (CCR7^+^CD45RA^+^) as the origin and computing pseudotime weights, we found that the protein expression of memory and effector markers increased with pseudotime following a transition from naive to memory and effector T cell states. In corroboration, we also computed CoGAPS patterns, identifying 3 patterns that were annotated as naive, memory, and effector based on the pattern weights of key markers (Figure 5A). These protein-expression patterns also corresponded well with their respective topologies when overlaid on the CyTOF UMAP (Figure 5B).

To determine the sensitivity of these findings with the analysis method selected to generate the metrics, we compared these results with those of other trajectory inference methods recommended by dynverse, a tool that compiles trajectory inference methods and suggests those most appropriate for the data and trajectories of under study (15). Using the PDAC Tc cell population across patients from both time points, we evaluated the correlations among the results based on Monocle3, Waterfall, Comp1, Tscan, and Scorpius (16, 29–32), confirming that pseudotime weights quantifying memory to effector transitions significantly correlated with each other (Spearman’s, *P* < 0.001) and were consistent across methods (Figure 5C). We also compared these weights from the different trajectory inference methods to the naive, memory, and effector CoGAPS patterns and confirmed that the memory pattern was positively correlated with pseudotime (Spearman’s *r*, 0.16 to 0.84, *P* < 0.001), whereas the naive pattern was negatively correlated with pseudotime (Spearman’s *r*, –0.19 to –0.79, *P* < 0.001) and the effector pattern was overall less correlated with pseudotime across methods (Spearman’s *r*, –0.018 to 0.01, *P* < 0.001).

The availability of pre- and posttreatment profiles in this data set enabled us to evaluate whether these T cell metrics can reflect the changes in T cell states resulting from therapy. Overall, we found that Th cell pseudotime weights significantly increased after 7 weeks of therapy (2 treatments of vaccine plus ipilimumab 3 weeks apart) in these patients (paired *t* test, *P* < 0.0001), suggesting a positive shift in Th cell activation or antigen experience (Figure 5D). This was also observed with CoGAPS-inferred Th and Tc cell memory pattern weights, which also increased after 7 weeks of treatment (*t* test, *P* < 0.01, Th cell; *P* < 0.05, Tc cell) (Figure 5, E and F). To test the potential of associating CyTOF-based T cell metrics with patient outcomes, we used a Cox proportional hazards model to compare overall patient survival of binarized groups defined using the median of T cell metrics means at baseline. Even with a small sample size, our analysis demonstrated that patients with higher Th cell effector pattern weights exhibited significantly increased overall survival (Cox, *P* < 0.05; HR, 5.36) (Figure 5G). Altogether, these results suggest that our approach can effectively quantify shifts in T cell states during immunotherapy and lead to correlates of clinical outcomes.

### Continuous CyTOF metrics can be projected across data sets to efficiently query patterns associated with clinical features of disease and patient outcomes.

To further extend the utility of CyTOF workflows, particularly in the setting of analyzing disparate immunotherapy studies, we next sought to determine whether other data sets can be projected into the previously learned functional states from a CyTOF data set. Leveraging the integrated CyTOF signatures found in our PDAC study, we employed transfer learning of those metrics to 2 additional independent CyTOF data sets using projectR, our transfer learning tool to project cell state patterns learned from matrix factorization to query their occurrence in new data sets (13, 33, 34).

**Figure 6:**
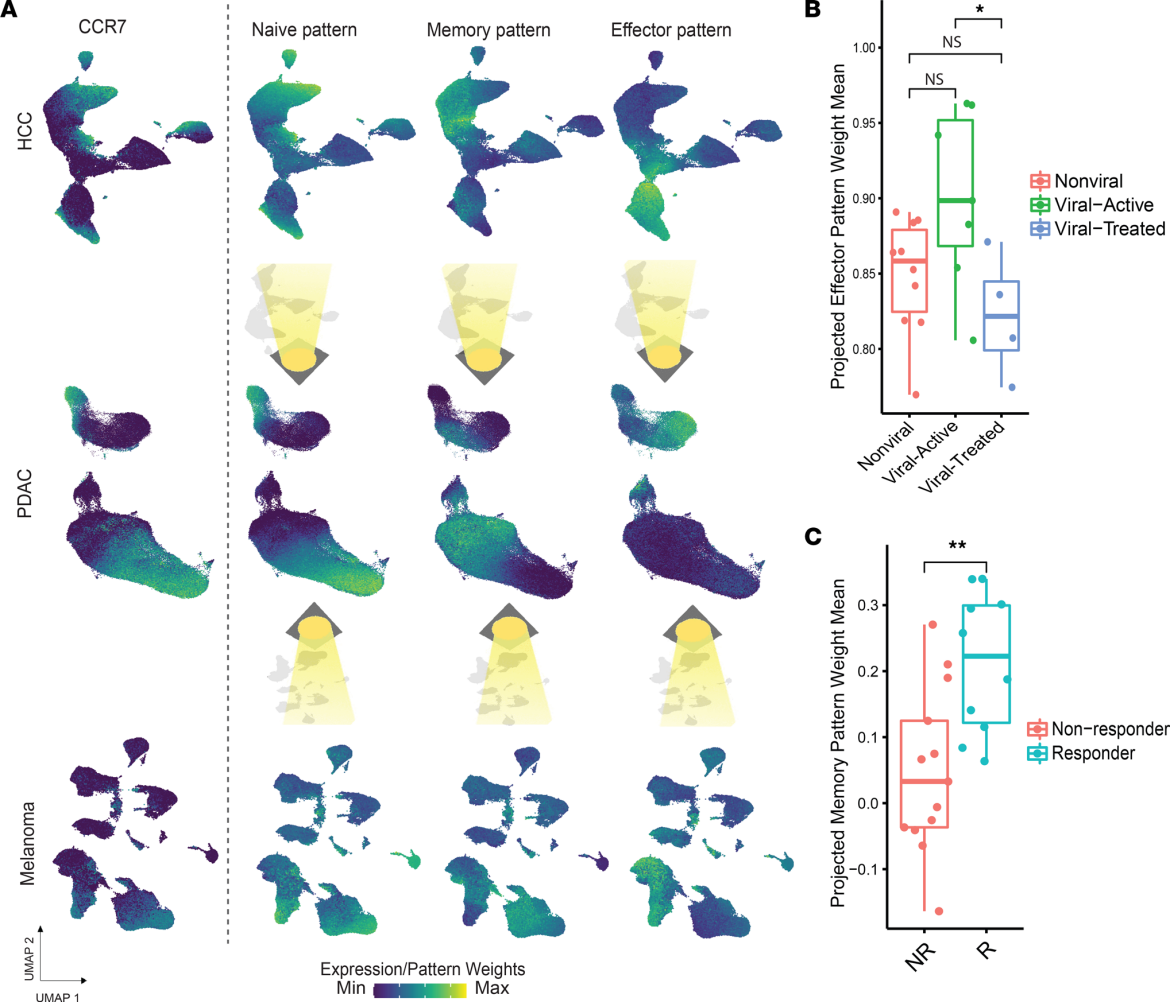
CyTOF integrated metrics can be projected across distinct patients, diseases, and immunological scenarios to annotate newly generated data sets and query proteomic outcomes-associated signatures. (**A**) CCR7 expression. NMF patterns from the PDAC CyTOF data set projected onto different cohorts of patients with HCC treated with nivolumab and patients with melanoma treated with ipilimumab. (**B**) Projected memory pattern weights in CD8^+^ T cells by response of patients with melanoma to ipilimumab, showing significantly increased means in responders at baseline. (**C**) Projected effector pattern weights in CD8^+^ T cells by patients with HCC disease etiology, showing significantly different means between treated and untreated viral status. Statistical significance was determined by group mean comparison *t* tests. Statistically significant *P* values adjusted for multiple comparisons (Holm-Bonferroni) are shown as follows: *P < 0.05; **P < 0.01.

First, we projected a CyTOF data set we previously generated with the same antibody panel but in patients with HCC treated with nivolumab who presented either with nonviral etiologies, treated hepatitis infections, or active hepatitis infections (35) into the CoGAPS T cell patterns from the PDAC study. This data set allowed us to explore whether T cell profiles affected by a different immunologic context, i.e., the different viral histories across patients, could be compared by transfer learning of integrated T cell signatures. Using the CoGAPS patterns learned in the PDAC CyTOF data set led to the rapid characterization of T cells in the HCC cohorts without the need for any manual annotations (Figure 6A). Upon transfer learning, we observed that the resulting effector pattern weights in the projected target data were significantly increased in CD8^+^ T cells from patients with HCC with active viral hepatitis infections compared with those with prior hepatitis infections who were treated effectively with antiviral therapies (*t* test, *P* < 0.05*)* (Figure 6B).

Second, we projected a publicly available CyTOF data set from patients with melanoma treated with ipilimumab (5). Because this data set was generated with a different antibody panel for T cell phenotyping and contained annotations of clinical responses to ipilimumab, the data set was particularly useful for testing whether transfer learning using the T cell signatures could tolerate nominal differences in the antibody panel and whether they can associate with clinical outcomes in another study. Again, using the CoGAPS patterns from the PDAC CyTOF data set (Figure 6A), we were able to observe that projected CD8^+^ T cells from patients with melanoma who clinically responded to ipilimumab therapy (*t* test, *P* < 0.01) (Figure 6C) were significantly increased in the previously learned memory pattern. This was consistent with the interpretations from the study based on analyzing discrete cell subtype abundances, showing higher frequencies of CD8*^+^* T effector memory subsets (5). In a separate validation analysis, we repeated CoGAPS learning of T cell patterns directly from these data sets and found signatures consistent with naive and effector phenotypes and canonical single protein markers (Supplemental Figure 3 and Supplemental Figure 4). Altogether, these results demonstrate a practical and integrated workflow using CyTOF profiling for empowering immunologic cross comparisons, particularly in the setting of multiple immunotherapy clinical studies.

## Discussion

This study provides a framework that can be used to apply single-cell mass cytometry analysis to a wide range of clinical immunology settings. As demonstrated by its use in T cell profiling (21) and understanding epithelial-mesenchymal transition states (22), trajectory inference has previously been applied for the analysis of high-parameter cytometry data sets (36). In this study, we implemented distinct approaches for CyTOF analysis: trajectory inference, clustering, and dimension reduction through NMF. All of these algorithms employ unsupervised learning to infer cellular phenotypes directly from the data, with clustering learning cellular subtypes, trajectory inference monotonic transitions between cellular phenotypes, and NMF distinct groups of continuous patterns distinguishing sets of phenotypic transitions. With these methods, we derived integrated T cell metrics on a per-patient basis and evaluated the clinical utility of such metrics. We showed that patient-specific means of pseudotime and CoGAPS pattern weights from CyTOF data are confirmatory and correspond well with known biology without relying on individual markers for gating or discrete cell clusters for interpretation, which can help alleviate under- or overestimation of signal in single-cell data.

Specifically, using a CyTOF data set focused on CD8^+^ T cells from patients with HIV, we showed that CyTOF-derived pseudotimes, based on a T cell exhaustion trajectory and independently derived CoGAPS pattern weight distributions, can recapitulate previously established T cell biology in HIV infection. Furthermore, applying these approaches in the context of cancer immunotherapy, we demonstrated that CyTOF-based T cell metrics can reflect significant changes during active immunotherapy with the anti–CTLA-4 checkpoint inhibitor, ipilimumab, and the cancer-specific vaccine, GVAX, and associate with clinical endpoints. As an alternative to more conventional clustering and cell-type abundance analyses, which provide discrete measures of immune profiles, we showed the use of integrated, continuous metrics that incorporate multiple parameters simultaneously to interpret the immunological states in patients. We posit that this approach is complementary, overcoming the potential pitfalls related to cluster-based algorithms, such as the need for a detailed manual annotation process, identifying the most optimal number of metaclusters, or mutually exclusive assignment of cells to clusters.

Additionally, using 3 independent CyTOF data sets from patients with PDAC, HCC, and melanoma and our transfer learning software projectR (33), we demonstrated that CyTOF-derived integrated signatures with clinical correlations can be used for transfer learning across data sets, despite discordance in antibody panels and distinct disease contexts. We have previously illustrated the wide applicability of projectR in cross-data set and cross-species transfer learning of single-cell and bulk transcriptomic data in multiple biological contexts (34, 37). Our current work also implicates the utility of our approach in CyTOF-based translational applications, such as comparing immunological states of patients learned from NMF across several clinical trials, evaluating relatively uncommon events such as clinical response to immunotherapy in patients with PDAC, and validating specific T cell signatures in a data set of limited scale by projecting robust signatures identified in another well-established data set. Together, these examples highlight the ways in which our approach will improve the power and efficiency of CyTOF analysis used in clinical studies. Still, future extensions of projectR to enable transfer learning from pseudotime are needed in applications that leverage trajectory inference in place of NMF-based inference of cellular states. Altogether, the combination of machine learning via NMF and transfer learning applied onto CyTOF as described in this study can enable a machine-learning approach for clinical correlate inferences while offering enhanced portability of signatures learned by projecting onto different data sets and disease contexts. Beyond the examples presented in this study, many more biomedical applications for the translation of patient-specific distributions of continuous cell states inferred from CyTOF can be envisioned, as CyTOF panels are relatively easy to design and implement. Moreover, running CyTOF is relatively cost-effective on a per-cell basis (7), allowing for the testing of large numbers of patient cohorts that in turn enable larger-scale prognostic and/or predictive studies using T cell pseudotimes or CoGAPS patterns as stratifiers. The scalability and efficient turnaround of patient sample processing, data acquisition, and the demonstrated analysis pipeline offer the potential for real-time monitoring of patient responses to immunotherapeutics. Although the sample sizes of the individual data sets presented here are limited, future testing of this method on larger-scale data sets will serve as a further validation of its use in translational discovery.

There are, however, prerequisites for this analysis approach. To compare the degree of activation and/or antigen experience in T cells across patients, the panel of antibodies must include key markers to capture the extent of biological transitions. In other words, the panel design dictates the robustness of the T cell trajectory inferences. This is expected given that mass cytometry most typically measures 30–50 markers, the selection of which defines the biology that can be observed. Conversely, the panel design can be optimized to better resolve T cell states at specific points along the inferred trajectory. Furthermore, because CoGAPS patterns can denote functional states without inferred trajectories or the assumption of monotonic cell state transitions or reliance on a reconciled UMAP embedding, our use of pattern weights would still enable integrated comparisons across patients. Previous work leveraging these tools to analyze cell-state transitions that occur with immunotherapy in the context of higher-throughput scRNA-Seq data also establishes the value and applicability of this approach for biomarkers in that technology. These tools can also be applied to interpret new data sets developed from the advances in single-cell proteomics technologies that are allowing for characterization of ever larger panels (13). In conclusion, our study establishes the feasibility of utilizing continuous integrated CyTOF metrics as a streamlined tool for real-time patient monitoring and cross-cohort comparisons of patient immune responses to immunotherapy.

## Methods

### CyTOF data sets.

Samples related to HIV (25), PDAC (28), melanoma (5), and HCC (35) have all been previously described in detail. A list of mass cytometry antibodies, isotopes, and concentrations used for phenotyping as well as the methods for cell staining and data acquisition are provided in the respective referenced publications. For preprocessing of CyTOF data, randomization, bead normalization, and bead removal of data were performed on CyTOF software (Fluidigm; v6.7). Multiplexed data sets were also gated for viability and debarcoded into single samples as previously described (5, 25, 28, 35). Each preprocessed sample was exported as a separate FCS file for analysis.

### CyTOF pseudotime, clustering, and CoGAPS patterns analysis.

FCS files from each sample were subsequently read using FlowSOM (38) to extract protein expression matrices. Expression matrices from each patient were aggregated and arcsine transformed. To make computational time and memory feasible, expression matrices were sampled by 10,000 random cells per sample and incorporated into a Monocle3 CDS object. Markers used for barcoding were removed. In data sets where significant sample processing batch effects were observed, *Batchelor* was used to correct the embedding (39). After dimensional reduction using 10 principal components, each cell partition was annotated, and Tc and Th cells were processed for pseudotime analysis using Monocle3 or Dynverse and NMF using CoGAPS v3.8.0, with default parameters on all protein markers in each panel, except CD45 isotopes, which are used for immune cell enrichment barcoding (23, 24). In each inferred trajectory, the first node was selected as the start node for pseudotime ordering in the direction of naive T cell marker expression (CCR7^+^CD45RA^+^). Unsupervised clustering was performed using Leiden community detection embedded in Monocle3, with default parameters and resolution *= e*^6^. Projection of CoGAPS patterns was performed on protein expression matrices using the projectR package v.1.10 with default parameters (33). All analyses were performed in R v4.0.2. A schematic of the entire pipeline is provided in Supplemental Figure 5.

### Data and code availability.

All code is available at the following Github repository: https://github.com/Dimitri-Sid/CyTOFpatterns (branch, main; commit ID, b9f21cc44b1bfcd4c65728bad8550ddd617157c8).

### Statistics.

To compare pseudotime, cluster abundance, and NMF distributions among patients, we used 2-sample unpaired 2-tailed *t* tests to compare means between groups and paired 2-tailed *t* tests to compare differences in means between patients’ time points. ggpubr v0.4.0 was used to calculate 2-tailed *t* test *P* values and adjust them with the default Holm-Bonferroni method. We fitted a Cox proportional hazards model using the coxph function of the survival R package v3.12-13 to evaluate patient outcomes as a function of NMF pattern weights using the median of pattern weight means for stratification. Correlations were evaluated using a Spearman’s correlation test and FDR correction as part of the corr.test function of the psych package v 2.2.5. *P* values of less than 0.05 were considered statistically significant. All statistical analyses were performed in R version v4.0.2.

### Study approval.

Experiments and clinical trial data acquisition and processing were performed in accordance with protocols approved by the Institutional Animal Care and Use Committee and the Institutional Review Board of Johns Hopkins University.

## Author contributions

WJH, EJF, EMJ, GLSO, LD, HW, and DNS developed experimental and analytical methods, analyzed data, and wrote the manuscript. NEG, SC, SX, JL, WJH and MY contributed to CyTOF data generation. All authors reviewed and edited manuscript.

## Supplementary Material

Supplemental data

## Version 1 10/10/2022

Electronic publication
