# Supplementary material for: Integrated T cell cytometry metrics for immune-monitoring applications in immunotherapy clinical trials
Source: JCI Insight. 2022 Oct 10;7(19):e160398. doi: 10.1172/jci.insight.160398 (PMC9675468; doi:10.1172/jci.insight.160398)
Supplement: Supplemental data [file jciinsight-7-160398-s228.pdf]

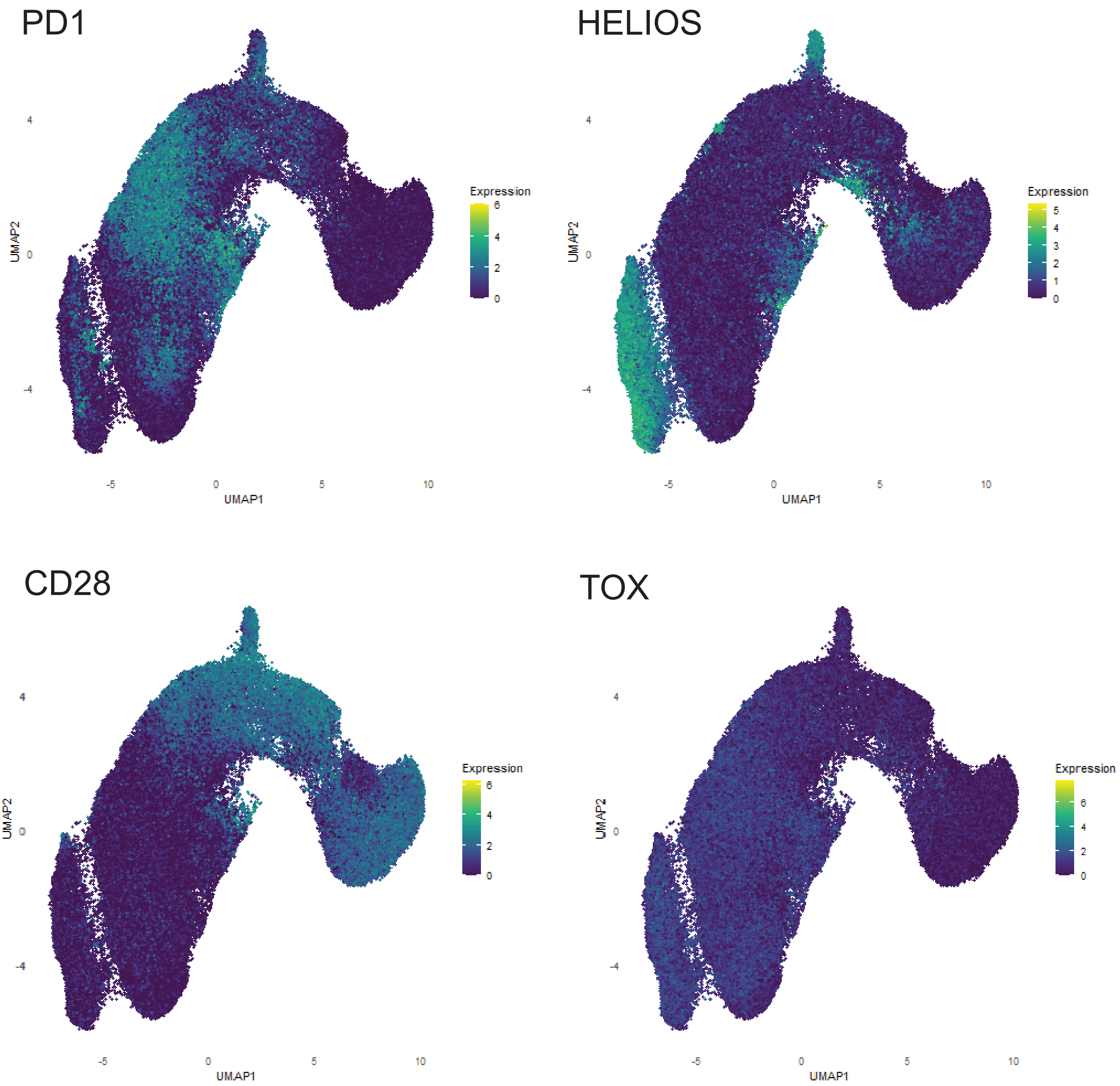

Supplemental Figure 1. UMAP overlayed with expression of exhaustion markers PD-1, HELIOS, TOX and activation marker CD28.

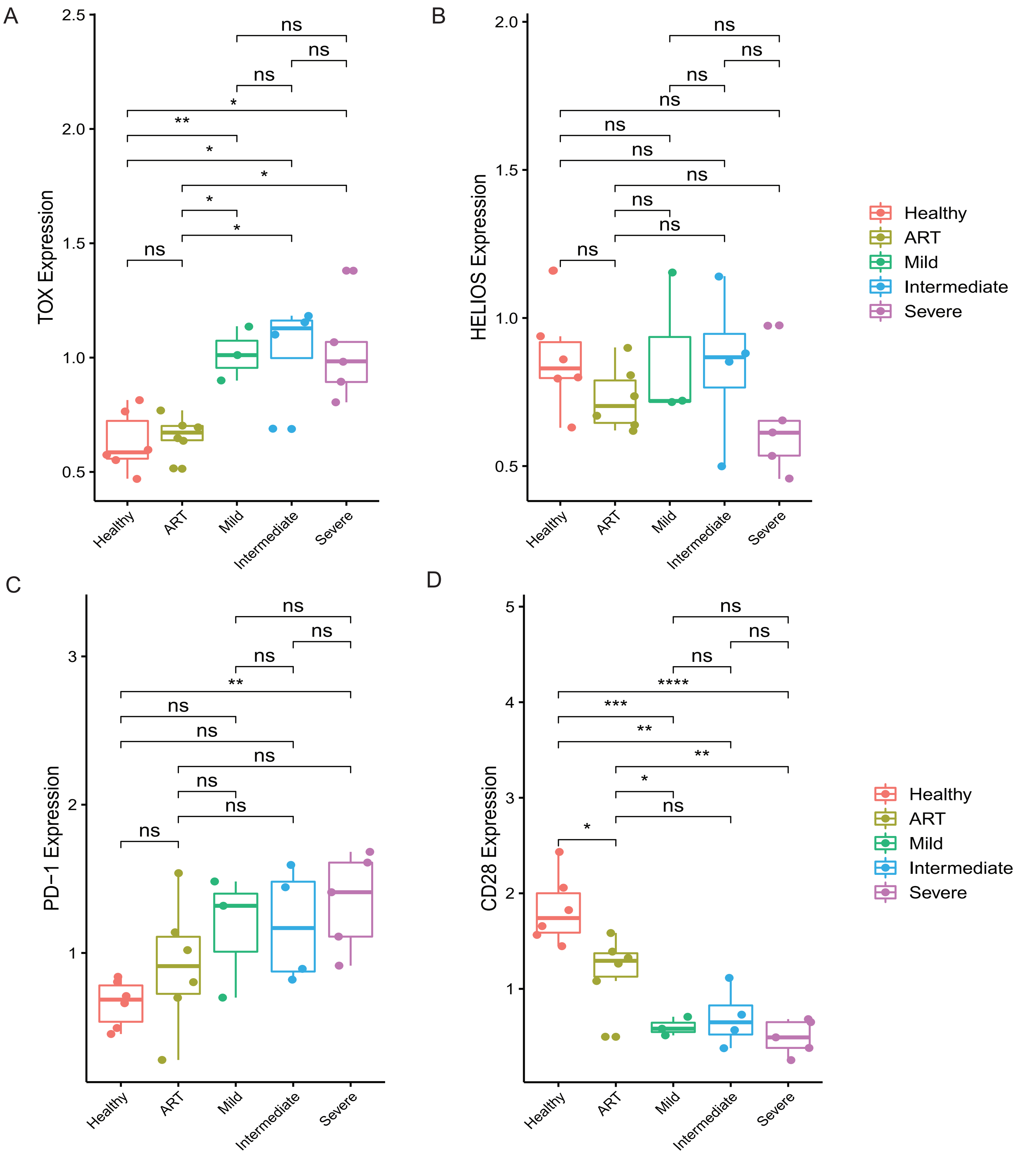

Supplemental Figure 2. Comparison of single marker expression across patients. Exhaustion markers TOX (A), HELIOS (B), PD-1 (C), and activation marker CD28 (D) average expression by patient. Statistical significance was determined by group mean comparison t-tests. Statistically significant P values are shown as follows: * *P < 0.05;* ** *P < 0.01;* *** *P < 0.001*.

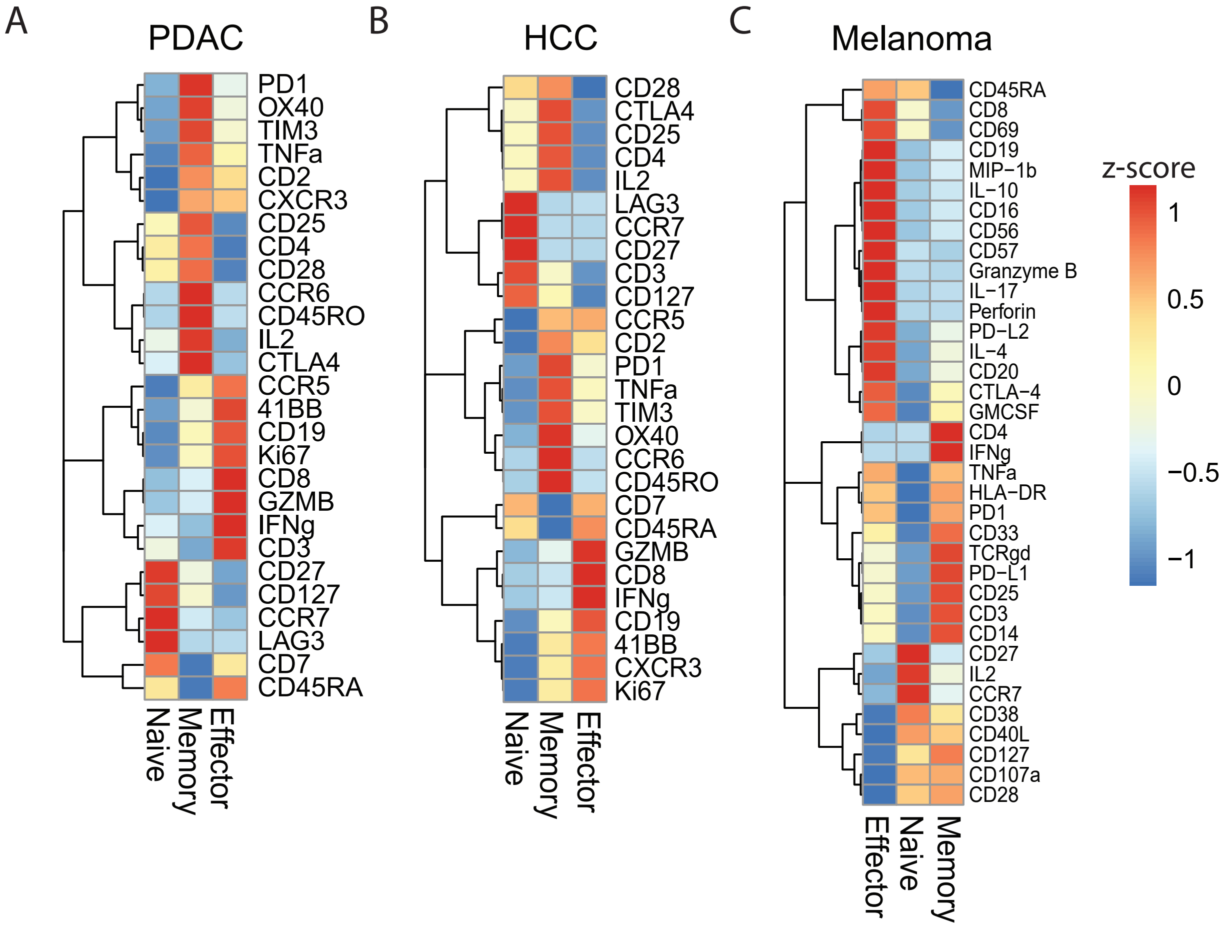

Supplemental Figure 3. Comparison of learned T cell NMF patterns in PDAC (A), HCC (B) and Melanoma (C) CyTOF datasets.

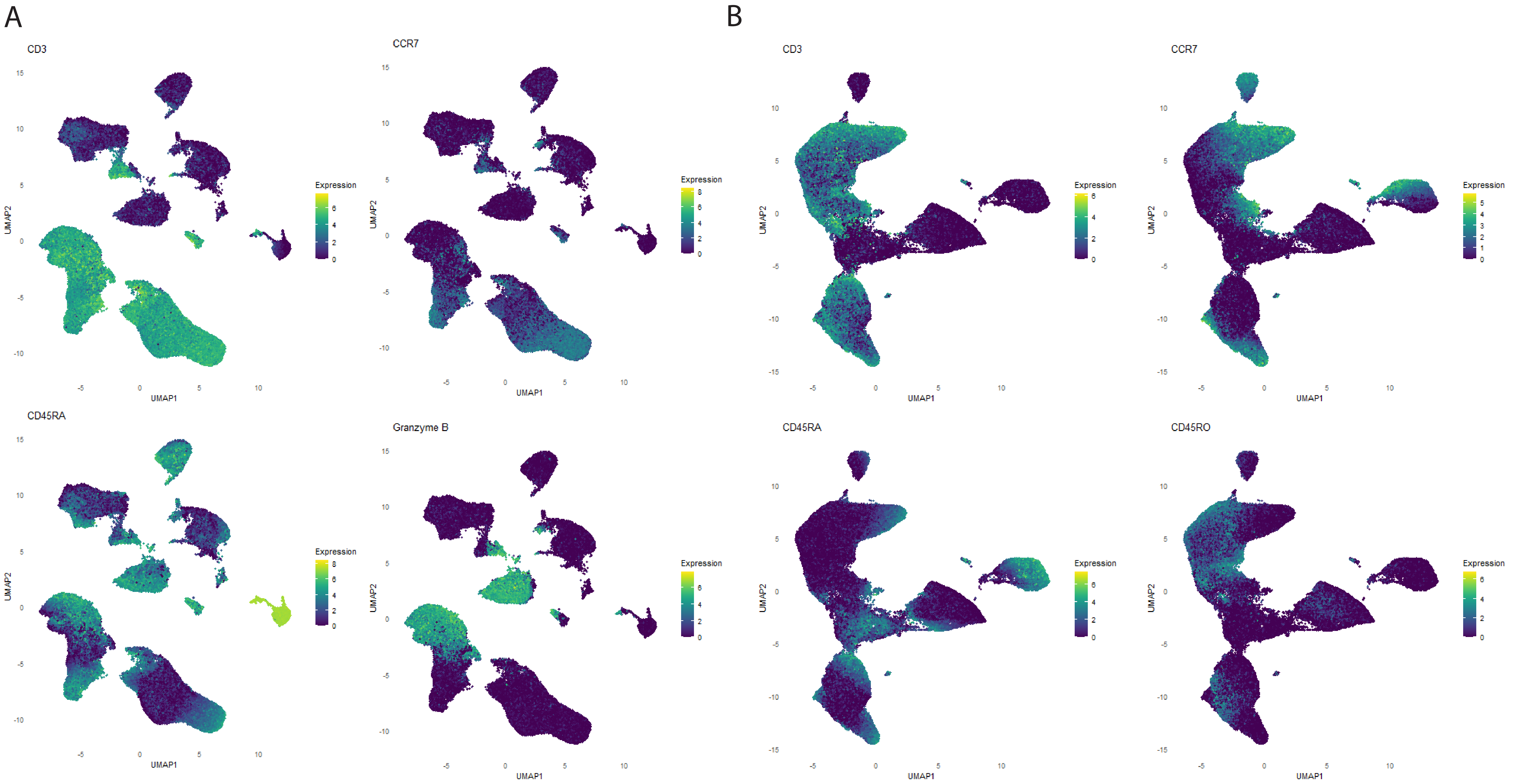

Supplemental Figure 4. UMAPs overlayed with expression of representative markers in Melanoma Hodi dataset (A) and HCC dataset (B).

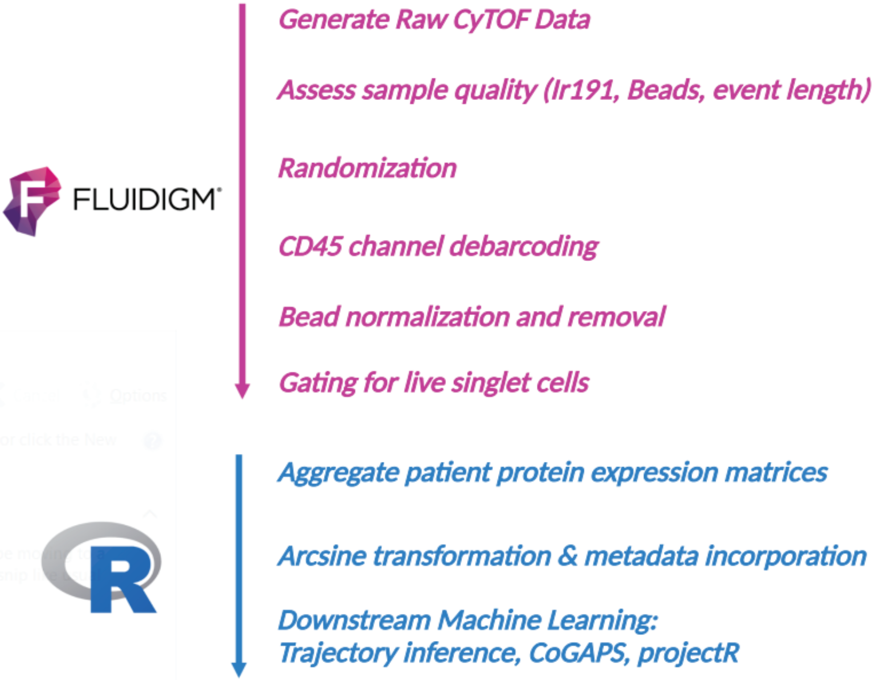

Supplemental Figure 5. Schematic of computational preprocessing and workflow.
